# HbA1c control in type 2 diabetes mellitus patients with coronary artery disease: a retrospective study in a tertiary hospital in South Africa

**DOI:** 10.3389/fcdhc.2023.1258792

**Published:** 2023-10-30

**Authors:** Lona Mhlaba, Dineo Mpanya, Nqoba Tsabedze

**Affiliations:** Division of Cardiology, Department of Internal Medicine, School of Clinical Medicine, Faculty of Health Sciences, University of the Witwatersrand and the Charlotte Maxeke Johannesburg Academic Hospital, Johannesburg, South Africa

**Keywords:** diabetes mellitus, HbA1c, coronary artery disease, glucose control, Africa

## Abstract

**Background:**

Type 2 diabetes mellitus (T2DM) patients with coronary artery disease (CAD) have an increased risk of recurrent cardiovascular events. These patients require optimal glucose control to prevent the progression of atherosclerotic cardiovascular disease (ASCVD). Contemporary guidelines recommend an HbA1c ≤7% to mitigate this risk. The aim of this study was to evaluate HbA1c control in T2DM patients with angiographically proven ASCVD.

**Methods:**

We conducted a cross-sectional, retrospective study on consecutive T2DM patients with acute and chronic coronary syndromes managed in a tertiary academic hospital in South Africa. Glycaemic control was assessed by evaluating the glycated haemoglobin (HbA1c) level measured at index presentation with acute and chronic coronary syndromes and during the most recent follow-up visit.

**Results:**

The study population comprised 262 T2DM patients with a mean age of 61.3 ± 10.4 years. At index presentation, 110 (42.0%) T2DM patients presented with ST-segment elevation myocardial infarction, 69 (26.3%) had non-ST-segment elevation myocardial infarction, 43 (16.4%) had unstable angina, and 40 (15.3%) had stable angina. After a median duration of 16.5 months (IQR: 7-29), 28.7% of the study participants had an HbA1c ≤7%. On multivariable logistic regression analysis, females were less likely to have poor glycaemic control (HbA1c above 7%) [odds ratio (OR): 0.42, 95% confidence interval (CI): 0.19-0.95, p=0.038]. Also, T2DM patients prescribed metformin monotherapy (OR: 0.34, 95% CI: 0.14-0.82, p=0.017) and patients with ST-segment depression on the electrocardiogram (OR: 0.39, 95% CI: 0.16-0.96, p=0.041) were less likely to have poor glycaemic control.

**Conclusion:**

After a median duration of 16.5 months, only 28.7% of T2DM patients with CAD had an HbA1c ≤7%. This finding underscores the substantial unmet need for optimal diabetes control in this very high-risk group.

## 1 Introduction

Type 2 diabetes mellitus (T2DM) is a global pandemic affecting approximately 537 million adults between 20-79 years (1). This figure is expected to rise to 643 million by the year 2030 and to rise even further to 784 million by the year 2045 (1). In South Africa, 10.1% of individuals older than 15 years have diabetes (2). This high prevalence poses significant health and socio-economic consequences (3). Diabetes mellitus leads to many diabetic-related complications, classified as micro and macrovascular complications. Microvascular complications include retinopathy, nephropathy, and neuropathy, while macrovascular complications encompass coronary artery disease (CAD), peripheral artery disease, and cerebrovascular disease (4).

Diabetes mellitus is a significant risk factor for CAD, causing accelerated atherosclerosis, resulting in severe and diffuse atherosclerotic cardiovascular disease (ASCVD) (5). In a meta-analysis involving 27,049 T2DM patients with at least one risk factor for cardiovascular disease, intensive glycaemic control reduced the risk of major adverse cardiovascular events by 9%, and the risk reduction was driven by myocardial infarction, while the risk of heart failure and stroke was unaffected (6). Furthermore, optimal glycaemic control has been found to slow the progression of coronary artery calcification and, therefore, atherosclerosis in asymptomatic diabetic patients without a history of CAD or stroke (7).

Multiple cardiovascular outcome trials evaluating the cardiovascular safety of novel anti-diabetic therapy have identified new diabetic therapeutic agents with proven cardiovascular benefits (8–12). It is now recommended that these agents with proven cardiovascular benefits be used as first-line therapy in T2DM patients with high and very high cardiovascular risk or established ASCVD (13). Many T2DM patients in low and middle-income countries (LMICs) are unable to access these organ-protective agents despite most diabetics in these regions failing to achieve adequate glycated haemoglobin (HbA1c) control on traditional agents alone (14). The aim of the study was to evaluate HbA1c control in T2DM patients with acute and chronic coronary syndromes, describe the management of these very high-risk patients using currently available therapies and determine the predictors of poor glycaemic control.

## 2 Materials and methods

### 2.1 Study design, study setting and data collection

A cross-sectional retrospective study was conducted on 1000 consecutive patients who presented with acute and chronic coronary syndromes in the Division of Cardiology at the Charlotte Maxeke Johannesburg Academic Hospital (CMJAH) between April 2017 and December 2019. The CMJAH is a state-owned tertiary academic hospital in Johannesburg, South Africa. Data was collected from the CMJAH catheterisation laboratory patient registry, which captures demographic data of all patients who undergo coronary angiography, and from the electronic health record system, which stores admission data of patients hospitalised in the cardiac intensive care unit and general cardiology wards.

**Figure 1:**
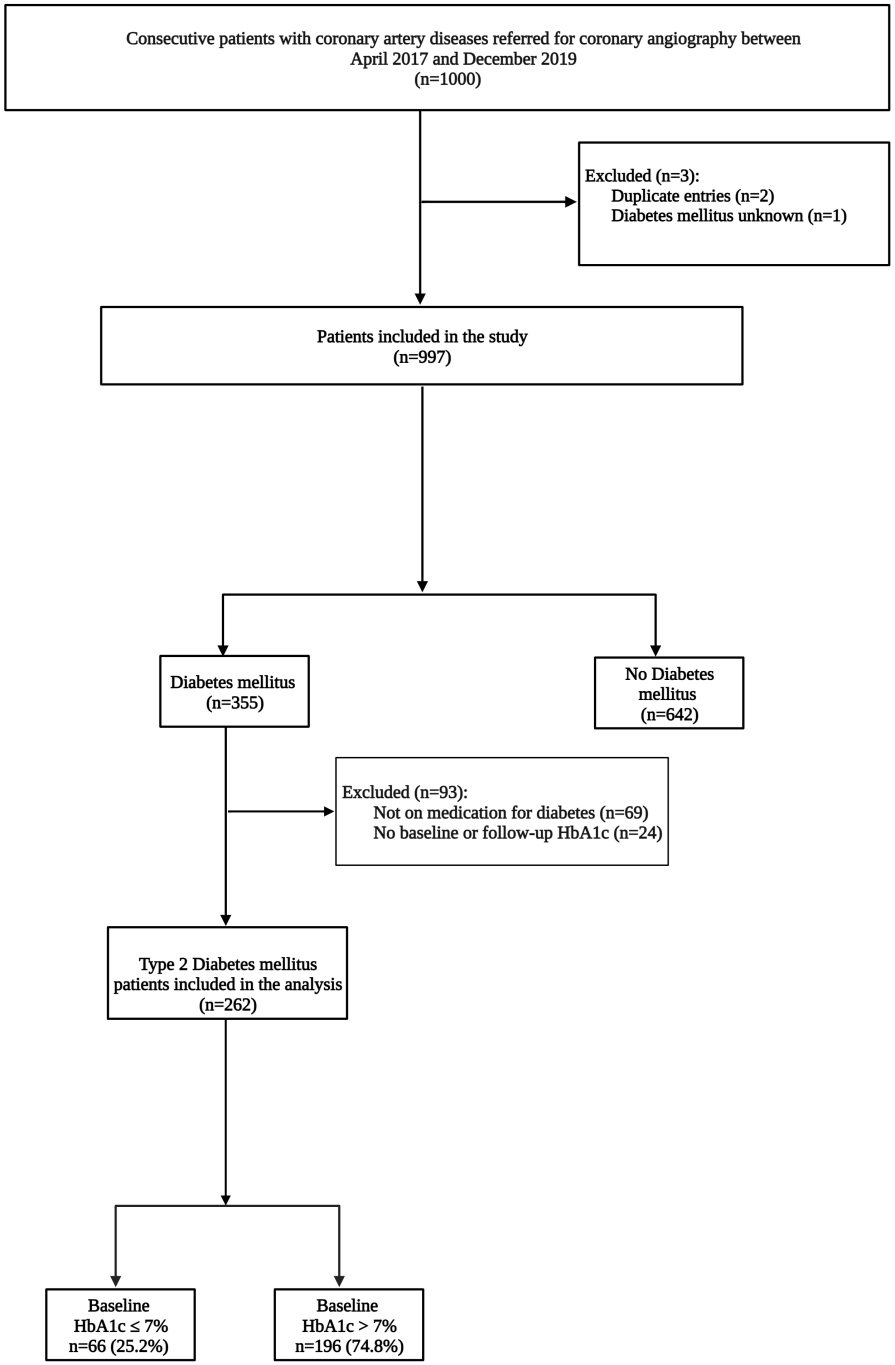
Flow chart outlining patient selection into the study.

Patients 18 years of age and older with T2DM and angiographically confirmed CAD were included in the study. Clinical parameters captured and analyzed as part of the study included patient demographics, risk factors for CAD, coronary angiography findings, clinical examination findings, laboratory parameters, electrocardiogram (ECG) and echocardiogram parameters, and medication. These clinical parameters reflected the patient’s status at the time of admission. To evaluate HbA1c control among T2DM patients, we assessed the baseline HbA1c measured when T2DM patients presented with coronary syndromes and the most recent HbA1c available on the National Health Laboratory Service electronic database. We defined optimal glycaemic control as an HbA1c ≤7% and poor glycaemic control as an HbA1c >7%. After excluding patients who did not meet the study inclusion criteria and those without baseline and follow-up HbA1c levels, the final cohort consisted of 262 T2DM patients (**Figure 1**).

### 2.2 Ethics approval and consentto participate

Permission to conduct the study was obtained from the University of the Witwatersrand Human Research Ethics Committee (clearance certificate number: M191191). Patients were not required to provide informed consent as this was a retrospective study.

### 2.3 Statistical analysis

Stata (version 17, College Station, Texas) was used for the statistical analysis. Categorical data is summarized as frequencies and percentages, and a Pearson’s chi-square test was used to compare categorical variables. Normally distributed continuous data is summarized using the mean and standard deviation (SD), and non-normally distributed continuous data is presented as the median and interquartile range (IQR). The Student’s t-test was used to compare normally distributed continuous variables, and the Wilcoxon rank sum test was used to compare continuous data with a non-normal distribution. Univariable and multivariable logistic regression analyses were performed to determine clinical variables associated with poor glycaemic control (HbA1c above 7%), and the results are presented as odds ratios (OR) with their corresponding 95% confidence intervals (CI). To select candidate variables for inclusion in the univariable logistic regression model, we selected variables with a p-value less than 0.1 after conducting the Student’s t-test, Pearson’s chi-square and Wilcoxon rank sum tests. We subsequently included variables in the multivariable logistic regression model with a p-value of less than 0.05 on the univariable analysis. A p-value of less than 0.05 represented statistical significance.

## 3 Results

The final study population comprised 262 T2DM patients, of which 188 (71.8%) were males. The mean age was 61.3 ± 10.4 years. At presentation with an acute or chronic coronary event, there were 148 (75.6%) to 196 (74.8%) T2DM patients with poor glycaemic control (HbA1c above 7%). Patients with a baseline HbA1c ≤7% were older than those with poor glycaemic control [63.6 ± 10.1 years (95% CI: 61.1-66.1) vs 60.5 ± 10.4 years (95% CI: 59.0-61.9); p-value= 0.0332]. The median systolic blood pressure was higher in patients with an HbA1c ≤7%, compared to those with an HbA1c above 7% [136 mmHg (IQR: 117-151) vs 124 mmHg (IQR: 112-142), p-value= 0.0121]. The median diastolic blood pressure was also higher in patients with an HbA1c ≤7%, compared to those with an HbA1c above 7% [85 mmHg (IQR: 75-97) vs. 78 mmHg (IQR: 71-88), p-value= 0.0205].

Among the 262 T2DM patients, 204 (77.9%) presented with Killip class 1 symptoms. Of these patients with Killip class 1 symptoms, 45 (68.2%) had an HbA1c ≤7% and 159 (81.1%) had an HbA1c above 7%, p-value= 0.029. Regarding the acute and chronic coronary syndrome presentation, 110 (42.0%) patients presented with ST-segment elevation myocardial infarction (STEMI). Of these patients with STEMI, 89 (80.9%) had an HbA1c above 7%. There were 69 (26.3%) patients with non-ST-segment elevation myocardial infarction, 43 (16.4%) with unstable angina and 40 (15.3%) with stable angina.

On the resting ECG at the time of admission into the hospital, 41 (15.7%) patients had ST-segment depression and 19 (46.3%) of these patients had an HbA1c ≤7%, while 22 (53.7%) had an HbA1c above 7%, p-value= 0.001. Coronary angiography revealed single vessel disease in 107 (40.8%) patients, double vessel disease in 92 (35.1%) and triple vessel disease in 63 (24.1%). Among the 63 patients with triple vessel disease, 51 (80.9%) had an HbA1c above 7%. The rest of the baseline demographics and clinical characteristics are depicted in **Table 1**.

**Table 1:** Baseline characteristics of patients with Type 2 Diabetes Mellitus and coronary artery disease at index presentation with coronary syndromes.

	ALL T2DM Patients n= 262	HbA1c ≤ 7% n= 66 (25.2%)	HbA1c > 7% n= 196 (74.8%)	p-value
Age (years)	61.3 ± 10.4	63.6 ± 10.1	60.5 ± 10.4	0.0332
Female, *n* (%)	74 (28.2)	26 (39.4)	48 (24.5)	0.020
Ethnicity				
Black, *n* (%)	56 (21.4)	14 (21.2)	42 (21.4)	0.970
White, *n* (%)	84 (32.1)	25 (37.9)	59 (30.1)	0.242
Indian/Asian, *n* (%)	102 (39.0)	22 (33.3)	80 (40.8)	0.281
Mixed Ancestry, *n* (%)	20 (7.6)	5 (7.6)	15 (7.7)	0.984
Risk Factors For CAD				
Previous CAD/CVA, *n* (%)	94 (36.6)	24 (37.5)	70 (36.3)	0.859
Hypertension, *n* (%)	198 (75.6)	52 (78.8)	146 (74.5)	0.482
Dyslipidaemia, *n* (%)	173 (66.0)	43 (65.2)	130 (66.3)	0.862
Chronic kidney disease, *n* (%)	40 (15.3)	13 (19.7)	27 (13.8)	0.247
Peripheral vascular disease, *n* (%)	11 (4.2)	5 (7.6)	6 (3.1)	0.114
Family history of CAD, *n* (%)	94 (35.9)	24 (36.4)	70 (35.1)	0.924
Smoker/ex-smoker, *n* (%)	150 (57.3)	37 (56.1)	113 (57.7)	0.821
Vital Signs				
Heart rate (bpm)	75 (64-90)	74 (66-89)	78 (69-96)	0.1628
Systolic blood pressure (mmHg)	124 (112-142)	136 (117-151)	124 (112-142)	0.0121
Diastolic blood pressure (mmHg)	80 (70-92)	85 (75.5-97)	78 (71-88)	0.0205
NYHA Functional Class				
NYHA 1, *n* (%)	132 (65.4)	33 (63.5)	99 (66.0)	0.740
NYHA 2, *n* (%)	61 (30.2)	13 (25.0)	48 (32.0)	0.343
NYHA 3, *n* (%)	6 (3.0)	4 (7.7)	2 (1.3)	0.020
NYHA 4, *n* (%)	3 (1.5)	2 (3.9)	1 (0.7)	0.102
Killip Class				
Killip 1, *n* (%)	204 (77.9)	45 (68.2)	159 (81.1)	0.029
Killip 2, *n* (%)	28 (10.7)	8 (12.1)	20 (10.2)	0.663
Killip 3, *n* (%)	9 (3.4)	3 (4.6)	6 (3.1)	0.567
Killip 4, *n* (%)	6 (2.3)	3 (4.6)	3 (1.5)	0.157
Biochemistry				
Troponin (ng/L)	1558 (204-5175)	1253 (28-4674)	1153 (158-3947)	0.8128
Estimated GFR (ml/min/1.73m²)	73.4 ± 27.5	67.5 ± 25.8	75.3 ± 27.9	0.0480
LDL cholesterol (mmol/l)	3.0 (2.3-3.8)	2.6 (2.0-3.4)	2.9 (2.1-3.6)	0.4844
Sodium (mmol/l)	139 ± 3.8	140 ± 3.2	138 ± 3.8	0.0001
Potassium (mmol/l)	4.4 ± 0.6	4.3 ± 0.6	4.5 ± 0.62	0.0679
ECG Parameters				
ST elevation, *n* (%)	84 (32.1)	13 (19.7)	71 (36.2)	0.013
ST depression, *n* (%)	41 (15.7)	19 (28.8)	22 (11.2)	0.001
Q waves, *n* (%)	76 (29.0)	13 (19.7)	63 (32.1)	0.054
T wave inversion, *n* (%)	33 (12.6)	9 (13.6)	24 (12.2)	0.768
Echocardiogram				
LVEF (%)	50 ± 14.1	51.1 ± 14.4	50.0 ± 14.0	0.6207
LVIDd (mm)	49.9 ± 8.9	50.6 ± 9.6	49.7 ± 8.7	0.5404
LVIDs (mm)	35.8 ± 9.5	37.7 ± 10.0	36.8 ± 9.5	0.5889

Bpm, beats per minute; CAD, coronary artery disease; CVA, cerebrovascular accident; ECG, electrocardiogram; GFR, glomerular filtration rate; HbA1c, glycated haemoglobin; LDL, low-density lipoprotein; LVEF, left ventricular ejection fraction; LVIDD, left ventricular internal diameter at end-diastole; LVIDS, left ventricular internal diameter at end-systole; NYHA, New York heart association; T2DM, type 2 diabetes mellitus.

Metformin was prescribed to 224 (85.5%) patients with T2DM. Among these patients, 140 (53.4%) were on metformin monotherapy, 41 (15.6%) patients were on metformin and insulin combination therapy, 40 (15.3%) were on metformin and sulphonylurea and three (1.1%) patients were prescribed metformin, sulphonylurea and insulin. **Table 2** depicts the rest of the medications prescribed to the T2DM patients.

**Table 2:** Diabetic medication prescribed to patients with Type 2 Diabetes Mellitus.

	All T2DM Patients n= 262	HbA1c ≤7% n=66 (25.2%)	HbA1c >7% n= 196 (74.8%)	p-value
Metformin, *n* (%)	140 (53.4)	48 (72.7)	92 (46.9)	<0.001
Sulphonylurea, *n* (%)	5 (1.9)	0 (0)	5 (2.5)	0.190
Insulin, *n* (%)	33 (12.6)	7 (10.6)	26 (13.3)	0.573
Metformin and sulphonylurea, *n* (%)	40 (15.3)	8 (12.1)	32 (16.3)	0.411
Metformin and insulin, *n* (%)	41 (15.6)	3 (4.5)	38 (19.4)	0.004
Metformin, sulphonylurea and insulin, *n* (%)	3 (1.1)	0 (0)	3 (1.5)	0.312

HbA1c, glycated haemoglobin; T2DM, type 2 diabetes mellitus.

**Figure 2:**
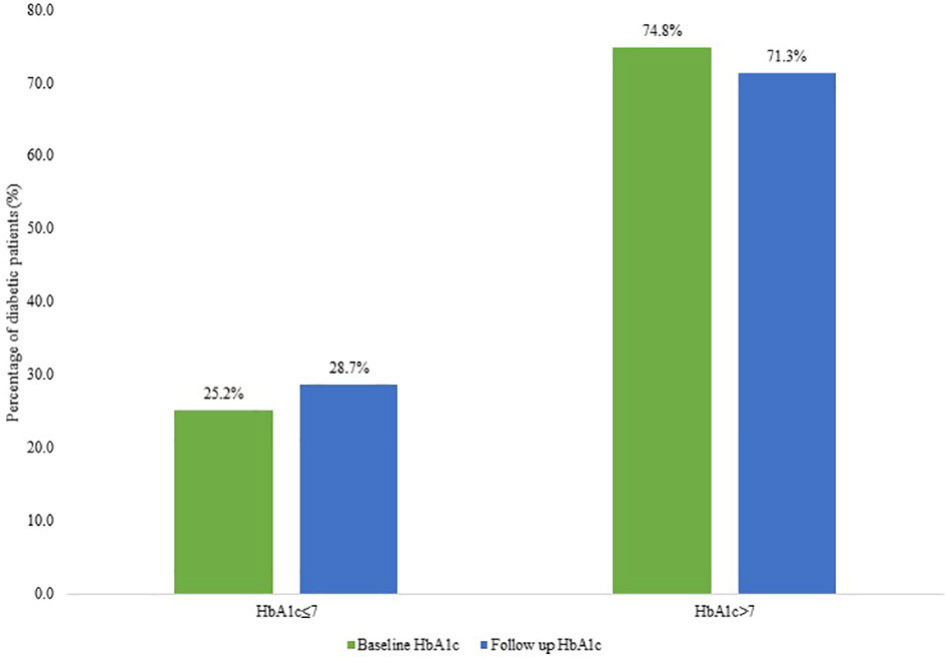
Graph showing baseline versus follow-up HbA1c in diabetic patients categorized as controlled diabetes mellitus (HbA1c ≤7) and poorly controlled diabetes mellitus (HbA1c >7%).

The proportion of diabetic patients with a baseline HbA1c ≤7% was 25.2% (95% CI: 18.4-31.5), and 74.8% (95% CI: 68.4-81.6) of T2DM had an HbA1c above 7%. After a median duration of 16.5 months (IQR: 7-29), 28.7% (95% CI: 22.2- 36.1) of T2DM patients had an HbA1c ≤7% and 71.3% (95% CI: 63.9-77.8) of patients had an HbA1c level above 7% (**Figure 2**).

Univariable and multivariable logistic regression analyses were conducted to identify clinical variables associated with poor glycaemic control (HbA1c >7%). On multivariable logistic regression analysis, females were less likely to have poor glycaemic control [odds ratio (OR): 0.42, 95% CI: 0.19-0.95, p=0.038]. Also, T2DM patients prescribed metformin monotherapy were less likely to have poor glycaemic control (OR: 0.34, 95% CI: 0.14-0.82, p=0.017). Furthermore, T2DM patients with ST-segment depression on the ECG were less likely to have poor glycaemic control (OR: 0.39, 95% CI: 0.16-0.96, p=0.041). However, patients in Killip class one were three times more likely to have poor glycaemic control (OR: 3.39, 95% CI: 1.26-9.12, p=0.015) (**Table 3**).

**Table 3:** Univariable and multivariable logistic regression analysis evaluating factors predisposing diabetic patients to poor glycaemic control (HbA1c above 7%) at index presentation.

	Univariable logistic regression			Multivariable logistic regression		
	OR	p-value	95% CI	OR	p-value	95% CI
Age	0.97	0.035	0.94-0.99	1.01	0.594	0.97-1.05
Female	0.50	0.021	0.28-0.90	0.42	0.038	0.19-0.95
Diastolic blood pressure	0.98	0.039	0.96-0.99	0.99	0.856	0.96-1.03
Systolic blood pressure	0.98	0.020	0.97-0.99	0.99	0.810	0.97-1.02
NYHA class 3	0.16	0.039	0.03-0.91	0.16	0.164	0.01-2.09
Killip class 1	2.00	0.030	1.06-3.76	3.39	0.015	1.26-9.12
Estimated GFR	1.01	0.050	1.00-1.02			
ST-segment depression	0.31	0.001	0.16-0.62	0.39	0.041	0.16-0.96
ST-segment elevation	2.31	0.014	1.18-4.54	1.53	0.393	0.57-4.09
Q waves	1.93	0.057	0.98-3.80			
Unstable angina	0.43	0.017	0.22-0.86	0.44	0.080	0.17-1.10
Sodium (mmol/l)	0.85	<0.001	0.78-0.92	0.89	0.065	0.78-1.00
Potassium (mmol/l)	1.55	0.069	0.97-2.49			
Metformin monotherapy	0.33	<0.001	0.18-0.61	0.34	0.017	0.14-0.82
Metformin and insulin	5.05	0.009	1.50-16.9	1.45	0.612	0.34-6.21

CI, confidence interval; GFR, glomerular filtration rate (ml/min/1.73m²); HbA1c, glycated haemoglobin; NYHA, New York Heart Association; OR, odds ratio.

## 4 Discussion

We conducted a cross-sectional, retrospective study on consecutive patients with T2DM and angiographically proven ASCVD. Considering that our study population had established ASCVD, glycaemic control should have been individualized based on the diabetes duration, life expectancy and burden of comorbidities (6, 15). It is recommended that in patients with a short life expectancy, such as elderly patients with a terminal illness or multiple comorbidities, the target HbA1c should be <8.5%. In individuals with a longer life expectancy, the target HbA1c should be < 7% (6, 15). In our study, we did not evaluate glycaemic control according to these criteria. We used HbA1c levels measured at the time of presentation with the acute or chronic coronary syndromes and during the most recent follow-up visit.

In our study, T2DM patients were treated with metformin, sulphonylureas and/or insulin therapy. However, despite using these traditional anti-diabetic agents, only 28.7% of these very high-risk T2DM patients achieved optimal HbA1c control after a median duration of 16.5 months. This finding underscores that glycaemic control in our study population is sub-optimal. This is comparable to data from other LMICs, where studies have shown similar results indicative of poor glycaemic control (16, 17).

Multiple landmark trials have demonstrated that intensive glycaemic control reduces microvascular complications in type 1 and type 2 diabetic patients (18, 19). However, strict glycaemic control did not yield a similar reduction in the occurrence of macrovascular complications. Some evidence suggests that the initial tight glycaemic control may reduce macrovascular complications in the long term (18, 20). These results are most likely attributed to the legacy effect or metabolic memory of tight glycaemic control (21). Furthermore, tight glycaemic control may lead to adverse effects. The Action to Control Cardiovascular Risk in Diabetes (ACCORD) trial evaluated tight glycaemic control in T2DM patients with established CVD. In this trial, patients were assigned to intensive treatment with a target HbA1c of less than 6% versus standard treatment with a target HbA1c between 7.0 and 7.9%. The average HbA1c achieved in the intensive therapy group was 6.4%, which was associated with increased mortality rates, hypoglycaemia and weight gain (22). Similarly, the United Kingdom Prospective Diabetes Study (UKPDS) trial evaluated tight glycaemic control in newly diagnosed T2DM patients and demonstrated similar adverse results (19). These deleterious effects may increase the risk of cardiovascular events and mortality (15, 23). Therefore, it is a first-line recommendation to avoid hypoglycaemia, especially in patients with CVD, to prevent the risk of subsequent events (24).

There is a substantial unmet need to control HbA1c levels and provide organ protection. Novel agents such as the sodium-glucose co-transporter 2 inhibitors (SGLT2-I) have demonstrated a reduction in major adverse cardiovascular events in T2DM patients with ASCVD (8–10). Similar results have been shown with some glucagon-like peptide 1(GLP-1) analogues (12, 25, 26). The current use of these agents in our setting is limited due to their high cost. However, cost-effectiveness analyses investigating the potential impact of SGLT2-I and GLP-1 analogues in preventing ASCVD complications and reducing the overall cost burden on the healthcare systems are still lacking in LMICs (27, 28).

In a systematic review and meta-analysis of 220,689 T2DM patients, diabetes increased the risk of all-cause mortality, cardiovascular death and strokes (29). The risk of all-cause mortality and cardiovascular death has also been found to be higher in diabetic patients with prior myocardial infarction (MI) compared to non-diabetics with and without previous MI (30). Therefore, reducing the risk of subsequent cardiovascular events is imperative by instituting the early use of novel anti-diabetic therapy that provides organ protection (24). The 2023 European Society of Cardiology guidelines for managing CVD in patients with diabetes recommend that in patients with CAD and T2DM, SGLT2-I or GLP-1 analogues should be added to the treatment regimen, irrespective of HbA1c control (10, 31, 32). In those patients not on metformin, it is recommended that these agents should be instituted as first-line therapy (24).

Metformin was the anti-diabetic agent used by 85.5% of T2DM patients included in our study. This is in keeping with current diabetes clinical practice guidelines, which recommend metformin as first-line treatment in T2DM (33). Metformin monotherapy was prescribed to 53.4% of patients, and 15.6% of T2DM patients were on metformin and insulin combination therapy. Among these patients on metformin and insulin combination therapy, 92.7% had poor glycaemic control despite evidence suggesting that adding insulin to metformin monotherapy improves glycaemic control (34). Clinical inertia to intensify diabetic therapy is common in LMICs and significantly contributes to poor glycaemic control (35). Therefore, the poor glycaemic control in our study participants may have resulted from a lack of treatment intensification and a possible low level of commitment to use insulin therapy optimally.

In our study, poor glycaemic control was more common in younger patients. Several reasons could account for this occurrence. Firstly, younger patients are often less likely to commit to medication and self-management of diabetes practices (36). Secondly, younger T2DM patients tend to be less informed about their condition and are more likely to have misconceptions about their susceptibility to disease complications because of their younger age (37, 38). Furthermore, our study population consisted predominantly of male patients, probably because ASCVD is more common in males. This is in contrast with a report by the International Diabetes Federation, which revealed a similar prevalence of diabetes in men and women (1). Of the 196 patients with poor glycaemic control, only 74 (24.5%) were females. Also, this finding was confirmed on the multivariable logistic regression model, where females were less likely to have poor glycaemic control. However, other studies have shown that poor glycaemic control is more likely in women than men (39, 40). There are several hypotheses to explain this observation, including differences among men and women in glucose metabolism, body mass index, hormonal differences, and psychosocial factors (41). Clinicians managing females with T2DM in our setting likely managed the disease more aggressively in light of the emerging evidence suggesting that females are at a higher risk of poor glycaemic control than their male counterparts (39, 40). It is also plausible that in our study, females living with diabetes were more engaged with the optimal treatment and control of their diabetes than their male counterparts. This phenomenon has been noted in Northern Sweden, where men seemed to underestimate problems related to diabetes more than women (42).

Poor glycaemic control is often associated with high blood pressure (43). However in our study, patients with poor glycaemic control had lower systolic and diastolic blood pressure. These patients likely had extensive ASCVD, since poorly controlled diabetes is associated with more severe CAD (44). Furthermore, we found that 24.0% of T2DM patients had triple vessel disease. Among these patients, 80.9% had an HbA1c above 7%. Diabetes mellitus is often associated with more complex CAD with multi-vessel involvement. Increased HbA1c levels result in extensive coronary artery involvement (44). These patients generally require surgical revascularisation, translating to higher healthcare costs (45). This further underscores the need to optimise HbA1c control and offer medication that is proven to delay the progression of ASCVD, thereby decreasing the prevalence of complex CAD and the need for surgical interventions, ultimately leading to decreased total healthcare costs.

We also found that 42.0% of T2DM patients presented with STEMI, and 80.9% had poor glycaemic control. Also, 15.7% of T2DM patients had ST-segment depression on the ECG at the time of hospitalisation. Type 2 diabetes mellitus patients with poor glycaemic control are more likely to have severe atherosclerotic disease characterized by vulnerable plaques. Therefore, these patients are more likely to have acute plaque rupture and present with STEMI (44, 46). Furthermore, we found that ST-segment depression on ECG was independently associated with optimal HbA1c control. This is likely because patients with controlled diabetes have less atherosclerotic burden and more stable plaques than those with poor glycaemic control (47).

Given the poor HbA1c control found in our study population, other strategies, besides the early use of novel anti-diabetic agents, must be implemented. Patients with T2DM should be educated about critical aspects of self-management of their condition. These aspects include adherence to medication, dietary advice, physical activity, and self-monitoring of glucose levels. Self-management of diabetes is inadequate in most individuals residing in sub-Saharan Africa (48). Other strategies to optimise glycaemic control include frequent follow-up visits, decentralisation of care and implementation of nurse practitioner or community health care worker home-based visits.

This study highlights the complex interplay between glycaemic control, cardiovascular health, and various clinical factors in T2DM patients with established cardiovascular disease. Our findings emphasize the importance of personalised treatment strategies that consider gender, compliance to medication, and cardiovascular symptoms since newer agents that offer organ protection are not readily available in state-owned healthcare facilities. Furthermore, we provided valuable information on the burden of T2DM in patients with ASCVD and clinical parameters associated with poor glycaemic control. Also, our findings provide a framework for further research into factors influencing glycaemic control in this population.

Our study had several limitations. Patient data was reviewed retrospectively, and we excluded T2DM patients who did not have baseline and follow-up HbA1c results. This reduced the study sample size. Furthermore, this was a single-center study. Therefore, the generalisability of the findings may not apply to other centers. However, most patients seen in our hospital were referred from primary and secondary-level hospitals to our hospital for further specialist-driven management. Another limitation is that our study did not consider psychosocial factors and the duration of T2DM. Both these factors may have played a role in the glycaemic control of our study participants. We also attempted to evaluate patient compliance to anti-diabetic therapy. However, such information was rarely documented in patient files. Furthermore, although we assessed glycaemic control using the most recent HbA1c level, we did not collect and analyze other clinical parameters gathered during the corresponding follow-up outpatient visit. Despite these limitations, our study data provides real-world insights into the standard of diabetic control in a South African state-owned tertiary hospital for a very high-risk population of T2DM patients with established ASCVD.

## 5 Conclusion

In our study, only 28.7% of T2DM patients with CAD achieved an HbA1c ≤7% after a median follow-up duration of 16.5 months. The small proportion of T2DM with optimal glycaemic control highlights the importance of an individualized treatment approach and the need for better management strategies beyond HbA1c control in this very high-risk group of patients.

## Data availability statement

The raw data supporting the conclusions of this article will be made available by the authors, without undue reservation.

## Ethics statement

This study involved humans and was approved by the University of the Witwatersrand Human Research Ethics Committee (clearance certificate number: M191191). The study was conducted in accordance with the local legislation and institutional requirements. The ethics committee/institutional review board waived the requirement of written informed consent for participation from the participants or the participants’ legal guardians/next of kin because the study entailed a retrospective review of patient records.

## Author contributions

LM: Conceptualization, Data curation, Formal Analysis, Investigation, Methodology, Project administration, Validation, Visualization, Writing – original draft, Writing – review & editing. DM: Data curation, Formal Analysis, Investigation, Methodology, Software, Supervision, Validation, Visualization, Writing – review & editing. NT: Conceptualization, Data curation, Formal Analysis, Investigation, Methodology, Resources, Supervision, Validation, Writing – review & editing.
